# Physical Therapies in the Treatment of Post-COVID Syndrome: A Systematic Review

**DOI:** 10.3390/biomedicines11082253

**Published:** 2023-08-11

**Authors:** Juan Carlos Sánchez-García, María Rentero Moreno, Beatriz Piqueras-Sola, Jonathan Cortés-Martín, Antonio Liñán-González, Elena Mellado-García, Raquel Rodriguez-Blanque

**Affiliations:** 1 Research Group CTS1068, Andalusia Research Plan, Junta de Andalucía, School of Nursing, Faculty of Health Sciences, University of Granada, 18016 Granada, Spain; jsangar@ugr.es (J.C.S.-G.); bpiquerassola@gmail.com (B.P.-S.); antoniolg@ugr.es (A.L.-G.); emg2684@gmail.com (E.M.-G.); rarobladoc@ugr.es (R.R.-B.); 2 School of Nursing, Faculty of Health Sciences, University of Granada, 18071 Granada, Spain; mariiaren.mr@gmail.com; 3 University Hospital Virgen de las Nieves, 18014 Granada, Spain; 4 School of Nursing, Faculty of Health Sciences, Melilla Campus, University of Granada, 52005 Melilla, Spain; 5 Costa del Sol Health District, 29640 Fuengirola, Spain; 6 University Hospital San Cecilio, 18071 Granada, Spain

**Keywords:** physical exercise, post-COVID-19, fatigue, dyspnea, respiratory exercise

## Abstract

Introduction: Several days to months after diagnosis of SARS-CoV-2, 35% of patients have persistent symptoms in high incidence. This is referred to as post-COVID-19 Syndrome. There is a pressing need to find a way to help patients with the manifested symptoms. Objective: To show the different therapies that exist for post-COVID Syndrome and their efficacy. Methodology: A systematic review of the scientific literature was carried out. The data search was carried out in Scopus, PubMed, Cinahl, and Web of Science. Of the 106 articles found, 12 articles were obtained after applying the following eligibility criteria. Results: Interventions related to respiratory musculature and moderate intensity exercise both in supervised face-to-face sessions and in supervised home sessions led patients to a significant improvement in the symptoms presented. Conclusion: Physical therapies significantly reduce fatigue and dyspnea as well as other symptoms related to quality of life.

## 1. Introduction

As is well known, in March 2020, due to the SARS-CoV-2 coronavirus, a pandemic called COVID-19 was declared. It is known that almost 50% of SARS-CoV-2 patients with COVID-19 pneumonia can recover spontaneously from a functional point of view at 3 months [1]; however, it has been possible to observe the persistence of symptoms (11.5 ± 5.7 days), and sometimes up to 10–35% of patients have persistent symptoms after several days or months. In the same way it can happen with people who have been mildly ill, undiagnosed, or who may have late or persistent symptoms [2,3].

This syndrome, which is appearing, attracts attention because it refers to the sum of very diverse symptoms that last until after the confirmation of SARS-CoV-2 infection. When we speak of a syndrome in health, we refer to a “coexistence of several symptoms” [4,5,6]. Therefore, this syndrome will continue to exist even after the acute phase has ended and several symptoms are still present.

Several names have been coined for this syndrome among patients, such as persistent COVID or long COVID [6], but the one recommended by the WHO [7] for use is the term post-COVID-19, since it does not allude to any kind of durability or causality [3].

The symptomatology of this syndrome can be very heterogeneous. The prevalent post-COVID symptoms encompass fatigue, difficulty breathing, impaired sense of smell and taste, chest pain, muscle aches, as well as sleep and psychological disturbances [5]. This leads to a poor quality of life [2,3].

Studies, such as Simani et al. [8], have determined a prevalence rate of 5.8% to 43%. The symptoms of this syndrome related to physical and respiratory deterioration can affect the psychological health and, as a consequence, can condition the performance of physical activity [2]. All this affects the ability of individuals to achieve a full recovery, affecting the basic activities of daily living and even the return to work [9].

In order to find a correct approach to this syndrome, it is recommended to have a first consultation 4 weeks after the acute phase [10]. The assessment of each patient can be performed telematically or in person depending on the patient’s data. The use of scales and/or questionnaires will also help us for the subsequent comparison of the state of health and the follow-up of the evaluation, and will also allow us to unify criteria with the health professionals.

There is a study, in particular, that talks about the symptoms associated with post-COVID syndrome. It shows that there is a high incidence of the syndrome in question, exposing the imperative need to find a way to effectively and efficiently help patients with the aforementioned symptoms [11].

For this reason, a review of the literature is proposed to show the different therapies that exist for patients with post-COVID syndrome and to evaluate their efficacy.

## 2. Materials and Methods

### 2.1. Review Protocol

The methodology used for this report was a systematic review of the scientific literature published on physical therapies for the treatment of post-COVID syndrome, following the Preferred Reporting Items for Systematic Reviews and Meta-Analyses (PRISMA) [12] review protocol, which consists of a 27-point checklist of the most representative parts of an original article, as well as the process of elaboration of these sections.

### 2.2. Eligibility Criteria

Articles with randomised clinical trial (RCT) methodology and articles with case study methodology were selected. The articles should be written after the COVID-19 pandemic was declared, January 2020, and should provide information on the modalities of therapies for the recovery of post-COVID syndrome in patients older than 18 years, without restriction in reference to the language of publication.

### 2.3. Sources of Information

This search was performed in the Scopus, PubMed, Cinahl, and Web of Science databases. In addition, a manual search was performed using reference lists of studies to find other relevant studies.

The structured language used was obtained by means of MeSH terms and health science descriptors (DeCS). The DeCs used were Post-Acute COVID-19 Syndrome and Physical Therapy Modalities, and the Boolean operators used were “OR” and “AND”.

### 2.4. Search Strategy

The following table (Table 1) shows the search strategy used for this work, the source, filters, and the date on which the search was performed.

### 2.5. Data Extraction Process

After carrying out the search strategy, the articles found were transferred to the Mendeley web application using the Mendeley web importer tool. They were then structured by folders, according to the databases through which they had been obtained, and duplicates were later eliminated.

The included studies were randomised clinical trials (RCTs) and cohort studies with the objective of showing therapies in post-COVID syndrome patients and evaluating their efficacy. The studies were published between 2020 and 2023. The title, abstract and keywords of each study were examined, and the inclusion and exclusion criteria were applied.

### 2.6. Data Collection Process and Data Collected

The following data were extracted from each article: men and women over 18 years of age who have had the disease, number of participants, type of physical exercise performed, duration of exercise, intensity, and whether it was supervised by professionals.

Section 3 shows the selection process of the articles in more detail.

### 2.7. Risk of Bias in Individual Studies

To carry out the methodological evaluation of the articles selected for this study, we proceeded to analyse the design, methodology and type of study of each article, with the aim of selecting the most specific methodological evaluation scale for each case.

Of the 13 articles, 4 were case studies, 1 was a cohort study, 7 were RCTs, and 1 was a quasi-experimental study.

The articles whose design was a case study were evaluated using the Single-Case Experimental Design (SCED) [13]. The SCED was constructed including 11 items, of which 10 are used to evaluate methodological quality and one for the use of statistical analysis.

The following table (Table 2) shows the results obtained after the methodological evaluation using the SCED scale [13].

For the articles whose methodology corresponded to a clinical trial, the scientific quality was evaluated using the PEDro scale [18]. This scale provides information on the clinical scientific evidence and scores it based on certain indicators, adding 1 point to each one if they are present and 0 points if they are not, giving a total score of 10 points. If the trial obtains a score between 9 and 10, it indicates that it is of very good quality; if it obtains between 6 and 8, it indicates good quality; if it is between 4 and 5, it indicates fair quality; and if it is less than 4, it indicates poor quality. In the case of the articles chosen for this systematic review, the values range between 6 and 9, receiving an average score of 8.30, which indicates that the average scientific quality is considered to be “good quality”.

The following table (Table 3) shows the results obtained after carrying out the methodological evaluation using the PEDro scale [18].

## 3. Results

**Figure 1:**
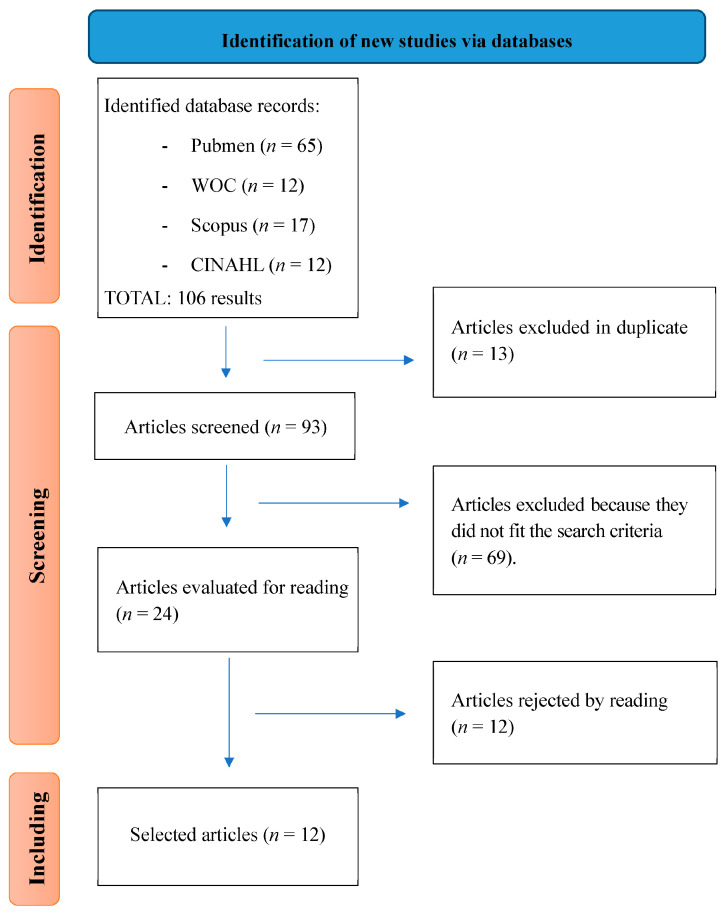
Flow diagram.

After applying the search strategy for articles in the different databases and applying the inclusion and exclusion criteria set out in the methodology, we identified 12 studies that we included in our review. Figure 1 shows the flow chart of the identified articles.

Taken together, the studies obtained highlight the efficacy of various therapeutic interventions to address the symptoms of prolonged COVID, encompassing physical and psychological well-being.

Overall, there were notable increases in physical function, with improvements in balance, muscle strength, and functional capacity, among others. Symptoms, such as fatigue and dyspnoea, decreased substantially in the intervention group compared to the control group. In addition, improvements in mental health and cardiovascular and pulmonary capacity were recorded. These results support the efficacy of exercise and rehabilitation strategies in the overall recovery of patients.

A summary of the results can be found in Table 4.

## 4. Discussion

The objective of this systematic review was to show the therapies that exist in patients with long-COVID and to evaluate their efficacy, and for this reason the study of the articles has been carried out.

This topic is closely related to the assessment of the systemic consequences of COVID-19, which is a broad field of research in which the assessment of respiratory function plays a key role. This was presented in the report by Pini et al. [26], where respiratory function was analysed 4–6 months after hospital discharge in these patients to study the negative consequences of COVID-19 pneumonia.

The results of this systematic review demonstrated that the exercise and rehabilitation strategies had a positive impact on multiple aspects of patients’ health, from physical function to mental health. These findings support the efficacy of the interventions implemented and suggest a pathway to improved recovery and well-being in people facing similar health challenges.

Most of the articles selected in the elaboration have been published in the year 2022, since we are dealing with a recent disease, namely COVID-19, and, above all, our objective concerns therapies against post-COVID syndrome. After analysing them, we can conclude that the selected articles have a generally good methodological level. We have been able to answer the main objective, since we have found different therapies for persistent COVID, such as exercises of moderate intensity [19,21], exercises for the respiratory musculature [15,20,22,23,24,25], electromagnetic field therapy [15,20,22,23,24,25], application of cutaneous electromagnetic nerve stimulation [9], and trigger point injections [17].

In the clinical guideline for long-COVID care, they recommend for fatigue a type of progressive exercise therapy tailored to the individual patient [27], information that we have been finding offers good results after completion [19,21]. In relation to dyspnoea, the guideline recommends respiratory exercise [20]. However, we cannot determine the efficacy of all studies as these have been based on a single case [9,14,17].

Several studies mention the improvement in the 6MWT test. Thanks to the controlled exercise, it was observed that men run a shorter distance when compared to women, with a significant increase for both [15,22,23].

Another improvement observed with controlled exercise was dyspnoea, which was shown to decrease significantly, with a decrease of approximately 80% in the control groups [15,21,22,24].

Depression and anxiety are a more subjective issue, since some studies show that there is a significant improvement in the control groups [24,25] but there is another that does not show a significant difference [16]. Despite that, it is observed that controlled exercise improves depression and anxiety.

Regarding articles that discuss electromagnetic field therapy [14], namely the application of electromagnetic nerve stimulation [11], it is shown that both women improved the sensation of fatigue, pain disappeared completely, and quality of life improved. On the other hand, the patient who received the trigger injections only manifested a complete disappearance of the pain [17]. It is necessary to qualify this aspect, as it is interesting to relate dry and wet needling with evident improvements in pain control in patients with post-COVID symptomatology. As shown in the case of Zha et al. [17]. It is true that this relationship can only be seen in this specific patient, so it is proposed as a new line of research derived from this study to substantiate this possible new treatment pathway.

One of the limitations that have been found is the poor adherence of study participants to the interventions [15,19,21,22,24] and the very small samples used [9,14,17].

Although there are several studies that demonstrate the efficacy of physical therapies, it remains to be determined whether other types of therapies or treatment would be effective against physical and psychological symptoms. And, above all, it is necessary to provide psychological and emotional help to these patients.

In terms of the limitations observed, more studies are needed, as the limitations are evident and may compromise the validity and reliability of the results. These limitations stem from sample sizes, the potential for bias, inadequate control of confounding variables and even the cross-sectional approach. Therefore, it is crucial to take these limitations into account when interpreting and applying the results of such studies to ensure accurate interpretation and appropriate use of their results in relation to physical therapies and prolonged COVID.

## 5. Conclusions

After searching the literature, we have found that moderate exercise and respiratory muscle exercises are beneficial for recovery from the most common symptoms of persistent COVID, namely fatigue and dyspnoea.

It can be concluded that, in cases where there was exercise control, patients have a considerable improvement in fatigue, depression and dyspnoea, among others.

However, there are still too few studies to be able to speak of the efficacy of certain therapies for the symptoms of long-COVID-19.

## Figures and Tables

**Table 1:** Search strategy details: source, filters, and search date.

Source	Search String	Filters	Date of Search	Items
WEB OF SCIENCE	(TS = (Post-Acute COVID-19 Syndrome)) OR TS = (“COVID-19 Syndrome, Post-Acute” OR “Post-Acute COVID-19 Syndromes” OR “Long Haul COVID-19” OR “COVID-19, Long Haul” OR “Long Haul COVID 19” OR “Long Haul COVID-19s” OR “Post Acute COVID-19 Syndrome” OR “Post Acute COVID 19 Syndrome” OR “Long COVID” OR “Post-Acute Sequelae of SARS-CoV-2 Infection” OR “Post Acute Sequelae of SARS-CoV-2 Infection” OR “Post-COVID Conditions” OR “Post COVID Conditions” OR “Post-COVID Condition” OR “Long-Haul COVID” OR “COVID, Long-Haul” OR “Long Haul COVID” OR “Long-Haul COVIDs”) AND (TS = (Physical Therapy Modalities)) OR TS = (“Modalities, Physical Therapy” OR “Modality, Physical Therapy” OR “Physical Therapy Modality” OR “Physiotherapy (Techniques)” OR “Physiotherapies (Techniques)” OR “Physical Therapy Techniques” OR “Physical Therapy Technique” OR “Techniques, Physical Therapy” OR “Group Physiotherapy” OR “Group Physiotherapies” OR “Physiotherapies, Group” OR “Physiotherapy, Group” OR “Physical Therapy” OR “Physical Therapies” OR “Physical” OR “Physical” OR “Neurological Physiotherapy” OR “Physiotherapy, Neurological” OR “Neurophysiotherapy”)	Articles and article reviews	6 March 23	12 results
PUBMED	((“Post-Acute COVID-19 Syndrome” [MeSH Terms]) AND (“COVID-19 Syndrome, Post-Acute” OR “Post-Acute COVID-19 Syndromes” OR “Long Haul COVID-19” OR “COVID-19, Long Haul” OR “Long Haul COVID 19” OR “Long Haul COVID-19s” OR “Post Acute COVID-19 Syndrome” OR “Post Acute COVID 19 Syndrome” OR “Long COVID” OR “Post-Acute Sequelae of SARS-CoV-2 Infection” OR “Post Acute Sequelae of SARS-CoV-2 Infection” OR “Post-COVID Conditions” OR “Post COVID Conditions” OR “Post-COVID Condition” OR “Long-Haul COVID” OR “COVID, Long-Haul” OR “Long Haul COVID” OR “Long-Haul COVIDs” [Title/Abstract])) AND ((“Physical Therapy Modalities” [MeSH Terms]) OR (“Modalities, Physical Therapy” OR “Modality, Physical Therapy” OR “Physical Therapy Modality” OR “Physiotherapy (Techniques)” OR “Physiotherapies (Techniques)” OR “Physical Therapy Techniques” OR “Physical Therapy Technique” OR “Techniques, Physical Therapy “OR” Group Physiotherapy” OR “Group Physiotherapies” OR “Physiotherapies, Group” OR “Physiotherapy, Group” OR “Physical Therapy” OR “Physical Therapies” OR “Therapy, Physical” OR “Neurological Physiotherapy” OR “Physiotherapy, Neurological” OR “Neurophysiotherapy” [Title/Abstract]))	Full text and in the last 5 years	6 March 23	65 results
SCOPUS	(((TITLE-ABS-KEY (“Post-Acute COVID-19 Syndrome”) OR TITLE-ABS-KEY (“COVID-19 Syndrome, Post-Acute” OR “Post-Acute COVID-19 Syndromes” OR “Long Haul COVID-19” OR “COVID-19, Long Haul” OR “Long Haul COVID 19” OR “Long Haul COVID-19s” OR “Post Acute COVID-19 Syndrome” OR “Post Acute COVID 19 Syndrome” OR “Long COVID” OR “Post-Acute Sequelae of SARS-CoV-2 Infection” OR “Post Acute Sequelae of SARS-CoV-2 Infection” OR “Post-COVID Conditions” OR “Post COVID Conditions” OR “Post-COVID Condition” OR “Long-Haul COVID” OR “COVID, Long-Haul” OR “Long Haul COVID” OR “Long-Haul COVIDs”))) AND (((TITLE-ABS-KEY (“Physical Therapy Modalities”) OR TITLE-ABS-KEY (“Modalities, Physical Therapy” OR “Modality, Physical Therapy” OR “Physical Therapy Modality” OR “Physiotherapy (Techniques)” OR “Physiotherapies (Techniques)” OR “Physical Therapy Techniques” OR “Physical Therapy Technique” OR “Techniques, Physical Therapy” OR “Group Physiotherapy” OR “Group Physiotherapies” OR “Physiotherapies, Group” OR “Physiotherapy, Group” OR “Physical Therapy” OR “Physical Therapies” OR “Therapy, Physical” OR “Neurological Physiotherapy” OR “Physiotherapy, Neurological” OR “Neurophysiotherapy”).))	Articles	6 March 23	17 results
CINHAL	(MH “Post-Acute COVID-19 Syndrome”) AND (MH “Physical Therapy+”)	Limiters: Refereed publications, expanders: Apply equivalent subjects and search modes: Boolean/Phrase	6 March 23	12 results

**Table 2:** Methodological evaluation results using SCED scale.

Author	Article	Numerical Score
Santos, et al. [9]	Musculoskeletal physiotherapy in physical sequelae of SARS-CoV-2 infection: A case report.	7/11
Wagner, et al. [14]	Successful application of pulsed electromagnetic fields in a patient with post-COVID-19 fatigue: A case report	4/11
Rausch, et al. [15]	The effects of Exercise Therapy Moderated by Sex in Rehabilitation of COVID-19	8/11
Daynes, et al. [16]	Early Experiences of Rehabilitation for individual sport-COVID to improve fatigue, breathlessness exercise capacity and cognition—A cohort Study	10/11
Zha, et al. [17]	Trigger point injections and dry needling can be effective in treating long COVID syndrome-related myalgia: a case report	6/11

**Table 3:** Assessment of methodology using the PEDro scale.

Author	Article	Numerical Score
Estebanez-Pérez, et al. [19]	The Effectiveness of a Four-Week Digital Physiotherapy Intervention to Improve Functional Capacity and Adherence to Intervention in Patients with Long COVID-19	6/10
Sharma, et al. [20]	Pulmonary Tele-Rehabilitation in Patients (Post COVID-19) With Respiratory Complications: A Randomized Controlled Trial	8/10
Jimeno-Almazán, et al. [21]	Rehabilitation for post-COVID-19 condition through a supervised exercise intervention: A randomized controlled trial	9/10
Sari, et al. [22]	Effects of Inspiratory Muscle Training in Patients with post-COVID-19	9/10
Okan, et al. [23]	Evaluating the Efficiency of Breathing Exercises via Telemedicine in Post-COVID-19 Patients: Randomized Controlled Study	9/10
McNarry, et al. [24]	Inspiratory muscle training enhances post-COVID-19 recovery: A Randomised controlled trial	8/10
Palau, et al. [25]	Effect of a home-based inspiratory muscle training programme on functional capacity in postdischarge patients with long COVID: The InsCOVID trial	9/10

**Table 4:** Summary of the conclusions of the results obtained.

Author	Sample	Type of Therapy	Results
Estebanez-Perez, et al. (2022) [19]	*n* = 32 (23 women and 9 men) Average age = 45.93 years	Digital physiotherapy for 4 weeks, with individual evaluation. One daily session of 45–40 min, three to five times per week. Walking, jogging, or swimming (20–30 min) for 3 to 5 sessions/week. Increasing strength training, exercising 1–3 muscle groups with a load of 8 to 12. Recommendation of ventilatory techniques to improve ventilation and mobility of the thorax.	After 4 weeks of intervention, a significant improvement was shown (*p* < 0.05). In the SPPB test (balance, gait speed, and chair support test) an improvement of 1.21 points was found. In the 1-STS test, an improvement of 3.50 points was obtained. There was an improvement in functional capacity, with high adherence rate and MCID values.
Santos, et al. [9]	*n* = 1 Age = 60 years	Therapy consisted of the application of transcutaneous electrical nerve stimulation, Cyriax deep transverse massage, stretching exercises, balance, coordination, and manual therapy, with Maitland passive kinesitherapy. Three times per week for 5 weeks.	Muscle strength improved from 2/5 to 4/5 on the Daniels muscle range test. Walking balance increased along with more coordinated movements. Fatigue and weakness disappeared. Patient can perform BADLs and IADLs normally again.
Wagner, et al. (2022) [14]	*n* = 1 Age = 55 years	Having noticed no improvement in previous therapies, he decided to use an electromagnetic field therapy, an ionic induction. Ten sessions of 30 min each, twice a week for 5 weeks. Patient placed in the supine position, 6 min are administered on the abdominal area, 3 min on the sternum, 6 min on the dorsal area, 6 min on the soles of the feet, and 6 min on the pelvic floor. The frequency used was 2.5 Hz for the dorsal area and 1 Hz for the rest.	The patient improved markedly with increased energy and complete disappearance of fatigue. There were improvements in the dimensions of mood, work, relationships, and enjoyment of life. There were no side effects except for transient neck pain.
Sharma, et al. (2022) [20]	*n* = 30 Average age = 18–55 years	Pulmonary telerehabilitation. The control group received conventional care and the experimental group received a therapeutic treatment protocol 4 days a week for 6 weeks. Exercises to reduce fatigue and shortness of breath.	Significant improvement in both groups, and there was also a significant difference between CG and EG in MBDS (*p* = 0.005 and *p* = 0.011) and VAS-F (*p* = 0.018 and *p* = 0.036). Therefore, it is concluded that the experimental group recovered more quickly. Women were more fatigued than men.
Jimeno-Alamazán, et al. (2022) [21]	*n* = 39 CG = 20 EG = 19 Age = 45.2 years	Eight-week supervised, personalised multi-component exercise program. Two days resistance training (3 sets, 8 repetitions of squat, bench press, dead weight, and bench pull) combined with moderate intensity variable training and one day of light intensity continuous training (30–60 min) for the experimental group. For the control group, aerobic exercise was recommended for 20 to 30 min, 5 days a week, at a tolerable intensity together with strength exercises in 3 sessions a week.	STS test, HSQ 50% 1 RM, estimated VO_2_max, and BP 50% 1 RM improved significantly in both groups. The most pronounced changes were dyspnoea (control vs. exercise: 83.3% vs. 5.4%, *p* = 0.003; V = 0.48) and fatigue (61.1% vs. 34.6%, *p* = 0.072; V = 0.30). In the exercise group, there was a progressive improvement in symptoms (94.7% vs. 72.2%, *p* = 0.063; V = 0.31), with patients being more likely to become asymptomatic (42.1% vs. 16.7%, *p* = 0.091; V = 0.28) than the control group. In cardiovascular parameters there was a loss in the main determinant of fitness in the control group (VO_2_max, 5.7% vs. −0.8%, *p* = 0.01) and final HR (−50.0% vs. −13.3%, *p* = 0.01). Lower limbs recovered in both groups when measuring the STS test (−22.7% vs. −20.7%).
Rausch, et al. (2022) [15]	*n* = 233 Women = 94, mean age = 61.50 years Men = 139, mean age = 61.69 years	Moderate therapy exercise, duration of 3 weeks. The 6MWT and a pulmonary function test were performed. They followed a standardised program which consisted of respiratory muscle training (3 sets of 10 breaths and 1 min rest), strength exercises, endurance training, and relaxation exercises.	Men received more respiratory strength exercises than women. No significant correlations were found between the number of respiratory muscle training sessions and lung function parameters (*p* > 0.05). In the 6MWT test, both men and women had statistically significant results (T (232) = −16.67; *p* < 0.001; d = 0.48). Men showed a shorter distance run compared to women (T (231) = −3.04; *p* < 0.01; d = 0.41). The improvement in ICmax was significantly higher in men (F (1227.46) = 8.93; *p* > 0.01; ω_2_ = 0.03). Men showed higher FVC before and after. The same was true for FEV1. Women showed a smaller difference with respect to FEV1 improvement. Significant reduction in FVC.
Sari, et al. (2022) [22]	*n* = 24 TG = 13 CG = 11 Age = 18–65 years	Inspiratory muscle training. Diagrammatic respirations, together with thoracic expansion and exercises to increase thoracic distensibility. A total of 10 repetitions, 3 sets per day. Resistance training to strengthen the quadriceps (squats and bridge exercises) for 6 weeks every day with 10 repetitions and 3 sets per day.	The 6MWT distance and the 30 s standing test increased significantly in TG (*p* < 0.001) and CG (*p* < 0.05). mMCR dyspnea scale significantly decreased in TG, from 10 to 2 patients with dyspnea in TG and from 7 to 6 patients with dyspnea in CG. Muscular strength of hand pressure increased significantly in TG.
Okan, et al. (2022) [23]	*n* = 49 IG = 26 CG = 26 Age > 18 years	Breathing exercises. In IG, 10 breathing exercises for 3 sessions per day, every day for 5 weeks. Light walking 20–30 min, 5 times a week. The CG had the exercises explained through a handout plus a recommendation for light walking.	The FEV1 and FVC values after the test in IG were significantly higher (95% CI: 2.921–8.771, 95% CI: 2.619–7.381 p_2_ < 0.001). Between IG and CG, the differences were not significant. In the MVV value, the IG had higher significance (97.54 ± 10.23). mMRC values were more significant in the CG. The 6MWT parameters were significantly higher in the IG.
Daynes, et al. (2021) [16]	*n* = 32	Rehabilitation for 6 weeks and 2 supervised days per week. Aerobic exercise, strength training of upper limbs and lower limbs. Educational meetings.	Thirty completed rehabilitations. All improved:ISWT by 112 m (*p* < 0.01) and 544 s (*p* < 0.01), FACIT by 6 points (pz 0.01), the EQ5D by 8 and MoCA by 2 (*p* < 0.01), and CAT by a score of 3 (*p* < 0.05). Anxiety and depression were not statistically significant.
McNarry, et al. (2022) [24]	*n* = 281 Age > 18 years	Inspiratory muscle training in 8 weeks. Intervention group and control group (standard care). Participants were trained to know how to use PrO_2_. Three sessions per week unsupervised. A total of 6 blocks of 6 breaths, interspersed breaks decreasing from 40 to 10 s in a maximum time of 20 min.	KBILD (dyspnoea and activities and psychological) had a significant improvement in GI. The GI had a good reduction in dyspnoea. It also significantly increased inspiratory muscle strength in the IG. Physical fitness and functional capacity increased significantly in the IG with an increase in VO_2_max.
Palau et al. (2022) [25]	*n* = 26 IG = 13 CG = 13 Age > 18 years old	Twelve weeks. IG training 2× week for 20 min each session with inspiratory muscle trainer applying a resistance of 25–30% of maximal inspiratory pressure. Diaphragmatic breathing will be instructed. The CG do not receive physiotherapy.	The IG mean VO_2_max was higher than that of CG (22.2 mL/kg/min, 95% CI: 21.3 to 23.2). For VE/VCO2 there was no significant difference between IG and CG (Δ −1.92; 95% CI: −4.69 to 0.85; *p* = 0.165). Significant improvement in depression/anxiety in IG. Non-significant improvement in mobility, self-care, and pain in both groups. IG had a significant improvement in MIP.
Zha et al. (2022) [17]	*n* = 1 Age = 59 years	Dry and wet puncture. At 6 months after presenting pain, WN was performed with 1% lidocaine without epinephrine with a 25-gauge needle and 1.5 inches, with four in the neck and shoulders, and one on each side of the triceps. Four sessions were performed. At 12 months, DN was tried with a 21-gauge 1-inch needle at 10 sites (4 in the neck and upper back region, 1 in the posterior triceps, and 2 in each calf). She carried out two sessions.	After several punctures, the patient improved markedly as he remained pain free 18 months later.

SPPB, Short Physical Performance Battery. 1-STS, 1-min sit-to-stand test. MCID, Minimal Clinically Important Difference. BADL, Basic Activities of Daily Living. IADL, Instrumental Activities of Daily Living. CG, control group. EG, experimental group. MBDS, Minimum Basic Data Set. VAS-F, Visual Analog Scale—Fatigue. HSQ, half squat exercise. 1 RM, one repetition maximum. VO_2_, volumen máximo de oxígeno. BP, bench press. HR, heat rate. 6MWT, 6 min walk test. ICmax, maximal inspiration capacity. FVC, forced vital capacity. FEV1, forced expiratory volume in the first second. TG, treatment group. mMRC, Modified Medical Research Council Dyspnea Scale. IG, intervention group. MVV, maximum voluntary ventilation. ISWT, Incremental Shuttle Walking Test. FACIT, Functional Assessment of Chronic Illness—Therapy Fatigue Scale. EQ5D, EuroQual 5 Domain. MoCA, Montreal Cognitive Assessment. CAT. COPD Assessment Test. PrO_2_, PrO_2_Fit Health (Device). KBILD, King’s Brief Interstitial Lung Disease. MIP, maximal inspiratory pressure. WN, wet needling. DN, dry needling.

## Data Availability

Not applicable.
